# The HSP90 Family: Structure, Regulation, Function, and Implications in Health and Disease

**DOI:** 10.3390/ijms19092560

**Published:** 2018-08-29

**Authors:** Abdullah Hoter, Marwan E. El-Sabban, Hassan Y. Naim

**Affiliations:** 1 Department of Biochemistry and Chemistry of Nutrition, Faculty of Veterinary Medicine, Cairo University, Giza 12211, Egypt; abdullah.hoter@vet.cu.edu.eg or abdo_hotar@yahoo.com; 2 Department of Anatomy, Cell Biology and Physiological Sciences, Faculty of Medicine, American University of Beirut, Beirut, Lebanon; me00@aub.edu.lb; 3 Department of Physiological Chemistry, University of Veterinary Medicine Hannover, Hannover 30559, Germany

**Keywords:** HSP90, GRP94, TRAP1, molecular chaperones, structure and function, pathophysiology

## Abstract

The mammalian HSP90 family of proteins is a cluster of highly conserved molecules that are involved in myriad cellular processes. Their distribution in various cellular compartments underlines their essential roles in cellular homeostasis. HSP90 and its co-chaperones orchestrate crucial physiological processes such as cell survival, cell cycle control, hormone signaling, and apoptosis. Conversely, HSP90, and its secreted forms, contribute to the development and progress of serious pathologies, including cancer and neurodegenerative diseases. Therefore, targeting HSP90 is an attractive strategy for the treatment of neoplasms and other diseases. This manuscript will review the general structure, regulation and function of HSP90 family and their potential role in pathophysiology.

## 1. Introduction

Serendipity played a decisive role in the discovery of what is now known as heat shock proteins (HSPs). In the laboratory of Italian scientist Ferruccio Ritossa [1], He examined flies, *Drosophila melanogaster*, that were left un-intentionally at an elevated temperature, and noticed a characteristic puffing in the chromosomes. He reasoned that this “puffing” phenomenon is due to the increased expression of specific proteins due to gene activation following heat stress. Hence, the name “heat shock proteins” came to light [2]. The discovery of heat shock proteins resulted in unraveling the heat shock response (also known as cellular stress response). This response is a common physiological process among almost all living organisms and includes alterations in the gene expression profile through increased HSP expression [3]. Overexpression of HSPs was found to promote cell survival and protect cellular proteins from the risk of damage or aggregation. In addition to heat shock, several factors increase the expression of HSPs including environmental and chemical factors, non-stress physiological causes and disease conditions [4].

HSPs are classified according their molecular mass, where HSP70 for instance meant subset of HSPs having molecular weight of 70-kDa. Due to the increasing numbers and confusing names of HSP members, which likely share a high degree of similarity in some cases, or differ greatly in other cases, there was a great need to classify these large overlapping proteins. Indeed, Kampinga and his colleagues [5] set a new letter/number based classification to simplify and unify the nomenclature of human HSPs. For instance, the previous names of HSP families—HSP70, HSP90, HSP110 and small HSPs (sHSPs)—which are still usable in the scientific community, were referred to as HSPA, HSPC, HSPH, and HSPB, respectively. Moreover, the members within each family were given numbers as HSPA1, HSPA2, HSPA6, HSPA8, etc. 

The members belonging to HSP90 protein family are highly conserved ubiquitous molecules with an approximate molecular weight of 90-kDa. They are molecular chaperones promoting the folding of de novo synthesized or incorrectly folded proteins, thus counteracting their aggregation. They exist in all living kingdoms except for archaea [6] and represent about 1–2% of the total mammalian cellular proteins under non-stress conditions [7]. In mammals HSP90 chaperones include members that are arranged into various cellular compartments. Two forms, HSP90 alpha and HSP90 beta, are located in the cytoplasm, GRP94 (94-kDa glucose-regulated protein) exists in the ER, and TRAP-1 (tumor necrosis factor receptor-associated protein 1) is present in the mitochondria. HSP90 proteins take part in essential cellular processes and regulatory pathways like apoptosis, cell cycle control, cell viability, protein folding and degradation, and signalling events. In addition, they induce the adaptive immunity by activation of antigen presenting cells and dendritic cells. Their prominent role in proteostasis, cell differentiation, and development is well recognized. Importantly, overexpression of HSP90 has been linked to many pathological conditions, like several types of cancer, viral infection, inflammation, and neurodegenerative diseases, suggesting a potential role in the pathogenesis of many diseases [7,8,9].

## 2. Classification, Structure, and Molecular Characteristics

### 2.1. Classification

According to the new guidelines for HSP nomenclature, the human HSP90 family includes five members that are categorized under the superfamily name HSPC [5]. The recent names together with the alternative old names of HSP90 members are listed in Table 1. 

HSP90 proteins can be classified according to their cellular localization, where cytoplasmic members include HSPC1 (HSP 90-alpha), HSPC2 (HSP 90-alpha A2), and HSPC3 (HSP 90-beta), ER resident member; HSPC4 or GRP94 (GP96), and mitochondrial localized member; and HSPC5 (TRAP1). Further classification of HSP90 is based on their expression pattern. For instance, Hsp90β is constitutively expressed, while Hsp90α is induced upon stress conditions [10,11].

### 2.2. General Structure of HSP90 Isoforms

The structural, biochemical, and molecular characteristics of HSP90 have been thoroughly reviewed [12,13,14]. We present in this manuscript a brief overview to provide the basic background for understanding HSP90 function under physiological and pathological conditions.

**Figure 1:**
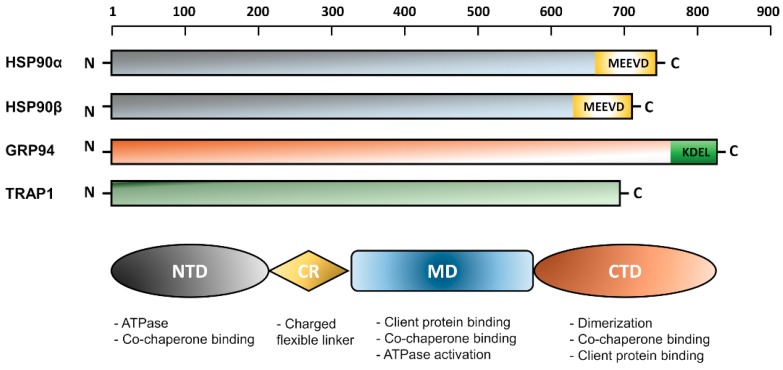
Domain structure of HSP90 family members in humans. The numbering 1–900 indicates the amino acid sequence. The lengths of HSP90α, HSP90β, GRP94, and TRAP1 are 732, 724, 704, and 803 amino acids, respectively. Below is shown a schematic representation of the domain structure of HSP90 isoforms together with the biological function of each domain.

The overall molecular structure of HSP90 homologues comprises three main conserved domains; N-terminal domain (NTD), C-terminal domain (CTD), and middle domain (MD) [7,15,16,17,18]. In eukaryotes, there is a variable charger linker domain which links the NTD to the MD [19]. Each domain within the HSP90 structure performs a specific function. The NTD binds to the ATP, hence, called the nucleotide-binding site. The CTD is responsible for protein dimerization and contains special motifs MEEVD or KDEL depending on the HSP90 isoform and its cellular location either in the cytoplasm or the ER. Although it has a divergent sequence among many eukaryotic organisms, the charged linker domain was found to be essential in the chaperone function, interaction, and flexibility [19]. A schematic representation of the HSP90 topology is shown in Figure 1.

#### 2.2.1. Molecular Characteristics of the Cytoplasmic Isoforms: Hsp90α and HSP90β

There are two main mammalian isoforms of HSP90 [17], presumably products of gene duplication [20], localized in the cytoplasm; the inducible form Hsp90α and the constitutive form HSP90β. Therefore, it is not surprising for the cytoplasmic HSP90 proteins to be highly homologous, with 85% sequence identity [21]. Although these cytoplasmic HSP90 isoforms have recently shown distinctive functions [17], most reviews do not distinguish them due to their remarkable structural and functional similarity. Therefore, the name HSP90 has been used for both HSP90 α and β unless indicated [18,22]. Studies dealing with the differentiation between cytoplasmic HSP90 isoforms, revealed that although both isoforms very often form dimers, HSP90α tends to dimerize frequently compared to HSP90β [17]. In addition, the α and β isoforms vary in specific regions along the protein sequence, suggesting diverse functions of the two isoforms.

Under physiological conditions, dimerization is essential for proper functioning of HSP90 [12]. Mammalian HSP90 is a phosphorylated dimer containing 2–3 covalently-bound phosphate molecules per monomer [18]. Moreover, the availability of other factors, such as divalent cations and nucleotides and elevated levels of HSP90 concentrations cause oligomerization of the molecule [18]. Structural studies, including sedimentation [23,24] and electron microscopy [25], revealed that dimeric forms of the protein have elongated structure [25]. As a hydrophobic chaperone, heat shock increases its hydrophobicity [26]. Another aspect is because HSP90 has an ATP binding site in its NTD, self-phosphorylation is likely to occur with profound conformational changes. Interestingly, although well known to have an ATP binding domain, HSP90 was found to have both ATPase and GTPase activity. In the presence of calcium, purified HSP90 was three-fold higher GTPase than ATPase [27].

##### The N-Terminal Domain (NTD)

Structurally, the N-terminal domain of human HSP90 appeared to be very similar to that of yeast HSPs [28]. It contains an ATP binding motif which shares high conservation with the GHKL (Gyrase, HSP90, histidine kinase, and MutL) superfamily and differs from the corresponding motifs of HSP70 or protein kinases [29]. The ATP-binding site is required for HSP90 ATPase activity necessary for the chaperone cycle and binding the HSP90 client proteins [29,30]. Of particular importance, NTD has been utilized as a major target for Hsp90 inhibitors such as geldanamycin (GA) and radicicol (RD). These are natural product inhibitors, which act by competing with ATP for binding the ATP-binding pocket in the NTD of HSP90.

##### The Charged Linker Region (CR)

Unlike prokaryotes, eukaryotic HSP90 has a short dynamic region, which links the NTD to the middle domain (MD). This domain is highly charged and has variable length and amino acid composition [31]. Since the eukaryotic HSP90 deals with a multitude of proteins, this charged linker region has been suggested to increase the flexibility and dynamicity to cope with the crowded environment of the eukaryotic cells [31,32]. There are contradicting reports about the role of the charged region in modulating HSP90 chaperone activity. While some studies suggest the charged region as a modulator of the chaperone activity [33], others neglect its potential influence on the HSP90 functions [34]. 

##### The Middle Domain (MD)

An important characteristic feature of the MD is that it modulates the HSP90 function by binding the γ-phosphate of ATP specified for the NTD thus modulating its ATPase activity [35]. Additionally, several studies demonstrated that this domain is implicated in binding co-chaperones like Aha1 and interacting with client proteins [36].

##### The C-Terminal Domain (CTD)

The CTD contains two key sites; one for calmodulin binding [37] and the other for HSP90 homodimerization [38]. In addition, it contains a nucleotide-binding site which opens following the occupancy of the N-terminal site and serves as allosteric regulator of the N-terminal ATPase activity [39]. Interestingly, the N- and C-terminal nucleotide binding sites exhibited differential specificities towards their ligands. The N-terminal binding site was found specific to adenosine nucleotides containing an intact adenine ring, like nicotinamide adenine dinucleotides and adenosine polyphosphate alarmones. On the other hand, The C-terminal binding site was much more unspecific by interacting with both purine and pyrimidine nucleotides. GTP and UTP nucleotides showed specificity towards the C-terminal binding site. Moreover, 2′,3′-*O*-(2,4,6-trinitrophenyl)-nucleotides (TNP-ATP, TNP-GTP) and pyrophosphate were able to bind the C-terminal binding site with no need for occupied N-terminal site [39]. Therefore, certain drugs like novobiocin (NB) [40] and epigallocatechin-3-gallate (EGCG) [41] could affect the function of HSP90 by targeting the CTD. It has been shown that CTD is included in the chaperone activity of HSP90 towards transcription factors and vesicular stomatitis virus G-protein [42]. Targeting the CTD by the AC-88 monoclonal anti-hsp90 antibody could hinder the interaction of HSP90 with many protein clients including steroid receptors and actin filaments [43]. Another characteristic feature of the HSP90 CTD is the existence of the MEEVD peptide sequence which binds the TPR-domain (tetratricopeptide-containing repeats) containing co-chaperones like HOP and immunophilins [44]. 

#### 2.2.2. Molecular Characteristics of the ER Isoform; GRP94

GRP94 (GP96) is the most abundant glycoprotein in the ER hence known as endoplasmin [45,46]. It is highly conserved among mammalian species. Interestingly the human GRP94 was found to be 100% identical to that of camel species [47]. GRP94 shares 50% homology with the cytoplasmic HSP90 [20]. However, several biochemical similarities exist between GRP94 and its cytosolic homologues including binding and hydrolyzing ATP. However, it binds to the HSP90 inhibitors geldanamycin and radicicol in a slightly different direction [48]. Nevertheless, being the major calcium binding protein in the ER and having specific limited protein clients are properties that clearly distinguish GRP94 from the cytosolic homologues [49].

GRP94 exists in three main conformations; the extended or chair-like conformation, the less extended conformation and the closed or twisted V conformation. The extended conformation confers better surface accessibility for binding client proteins as well as nucleotides [48,50,51]. Indeed, like HSP 90, the GRP94 is very dynamic where the presence of excess nucleotides, client proteins, and co-chaperones shift the conformation to the closed state [49].

Like HSP90, GRP94 has three main structural domains: the NTD, MD, and CTD. Notably, the N-terminal amino acid sequence differs from that of cytosolic HSP90 in both length and sequence [44]. This variability suggests heterologous conformational changes of HSP90 and GRP94. As an ER resident chaperone, GRP94 contains an N-terminal 21 amino acid sequence required for ER targeting. Another important difference between the cytoplasmic and ER HSP90 homologues is that in GRP94, the lid region, a term used to describe amino acid residues 100–121 in the cytosolic HSP90 and serves to couple ATP binding to N-terminal association, contains five additional residues between the two helices [48,50].

Compared to the cytosolic HSP90, the charged linker region is shorter, rich in lysine residues, more acidic, and contains many calcium binding sites [15]. Additionally, the charged linker domain modulates the conformational changes upon ATP hydrolysis and its deletion dramatically affect the GRP94 function and interaction with clients [52]. GRP94 binds calcium in high capacity, however, low affinity as it was known to have 11 low-affinity calcium binding sites and four higher-affinity Ca binding sites. Calcium binding can affect the molecular conformation and the activity of GRP94 [53]. Occupation of the Ca^2+^-binding site, located in the charged linker domain of GRP94, by calcium augments the binding of client peptides with the peptide-binding site in the N-terminal domain of the protein, thus regulating the capability of GRP94 to bind peptides [53].

The middle and C-terminal domains of GRP94 resemble those in HSP90 except for few differences. The dimerization interface is stricter in GRP94 and the CTD of GRP94 contains an extra 55 amino acids with a KDEL retention signal rather than MEEVD peptide at its end [15].

#### 2.2.3. Molecular Characteristics of the Mitochondrial Isoform; TRAP1

TRAP1 is the mitochondrial version of HSP90, which is localized primarily in the in the mitochondrial matrix and to a few extent in the inter-membrane space [54,55]. It shares high homology with the cytosolic counterpart reflecting 34% identity and 60% similarity [56] and have the same three domain architecture of HSP90: NTD, MD, and CTD. Similarities with other homologues exist where the NTD can bind ATP and it is vulnerable for inhibition by geldanamycin and radidcol. Notably, the binding affinity of TRAP1 to ATP is ten times higher than HSP90 [57,58]. Recently, X-ray diffraction studies showed that calcium can compensate for magnesium to support TRAP1 ATPase activity where the relative preference for magnesium or calcium is dependent upon the free ATP concentration [59]. In addition, calcium binding to TRAP1 induces cooperative ATP hydrolysis by the two HSP90 protomers, whereas magnesium binding causes non-cooperative ATP hydrolysis. Interestingly, heat shock can increase the expression of TRAP1 up to 200-fold [55]. This mitochondrial HSP90 has a 59 amino acid serving as a mitochondrial targeting sequence and is cleaved off upon import to the mitochondria [60]. Indeed, TRAP1 lacks both the C-terminal MEEVD motif and the charger linker domain connecting MD to the CTD which are present in its cytosolic homologue [61]. Moreover, certain co-chaperones, including p23 and Hop, are not required for its function [62].

## 3. Regulation of HSP90

There are different levels of regulation that control the function of HSP90; transcriptional regulation, posttranslational modification, and co-chaperones [12,63]. These factors can be summarized as follows. 

### 3.1. Transcriptional Regulation

Generally, the expression of HSPs is controlled by a subset of specialized stress related transcription factors named heat shock factors (HSFs). Binding of activated HSFs to their specific place, known as heat shock element (HSE), in the HSP promoter region stimulates the action of RNA polymerase on the coding region of the HSP gene [18]. Recent models propose that binding of HSP90 and HSP70 to HSF1 cause its inactivation. When chaperone function needed, dissociation of HSF1 occurs and HSP transcription is induced [63,64].

#### 3.1.1. Cytoplasmic HSP90

The gene encoding HSP90α is located on chromosome 14 while the gene encoding HSP90β is present in chromosome 6 [17]. Compared to the constitutively expressed HSP90β, HSP90α is highly induced by increased temperature. Several previous studies have demonstrated the promoter region and transcriptional regulation of HSP90 in yeast [18]. In human HSP90α, the promoter region contained a putative SP1 binding site and a serum-response element, together with multiple HSEs suggesting more complex regulation events [65].

#### 3.1.2. GRP94

In humans, the gene encoding GRP94 is known as *HSP90B1* and is located on chromosome 12. The regulation of GRP94 follows the same transcriptional regulation as in cytosolic HSP90. However, it is mainly induced by ER stress or overload rather than a simple heat shock. Like GRP78, the GRP94 promoter region has a CG/CAAT and a GC-rich sequence motif, which are essential for basal and induced expression of the genes [18]. Therefore, GRP94 regulation is associated with GRP78 regulation [15,49]. Decreased induction of GRP94 resulted in low induction of GRP78, while increased GRP78 induction caused enhanced GRP94 stimulation. 

#### 3.1.3. TRAP1

The gene encoding human TRAP1 is located on chromosome 16. Data are scarce about its transcriptional regulation. However, it appeared to follow the same general regulation of HSPs [60].

### 3.2. Regulation by Posttranslational Modification

Posttranslational modification (PTM) of HSP90 isoforms modulates their chaperone function in terms of accessibility of the binding sites [63]. The cytoplasmic isoforms; HSP90β and HSP90α undergo different PTMs including phosphorylation, acetylation, SUMOylation, methylation, ubiquitylation, and S-nitrosylation [12,66,67]. The endoplasmic reticulum form, GRP94, can be modified by glycosylation, phosphorylation and acetylation [49]. Additionally, the mitochondrial homologue TRAP1 is subject to phosphorylation [60,68]. However, other PTMs which affect cytosolic HSP90, like acetylation and nitrosylation, cannot be excluded for TRAP1 modification [60].

### 3.3. Co-Chaperones

Co-chaperones represent the most important layer of HSP90 regulation (Table 2). These molecules assist the chaperon proteins in performing their function. Several reports described many co-chaperones for cytosolic HSP90. A comprehensive up-to-date list of HSP90 co-chaperones can be obtained through (http://www.picard.ch/downloads/Hsp90interactors.pdf and [63,69]. Concrete data about GRP94 and TRAP1 co-chaperones are lacking because of their compartmentalization inside the ER and mitochondria [61,70]. However, recently MZB1 (pERp1) has been shown as a co-chaperone of GRP94 [71].

## 4. Client Proteins

### 4.1. HSP90

HSPs are biological catalysts. They mainly bind to their interacting partners through non-covalent binding where their substrates are commonly referred as clients [22]. As molecular chaperones, HSP90 isoforms have wide range of client proteins that are involved in numerous cellular pathways. Compared to other homologues, the cytoplasmic isoforms of HSP90 (Hsp90α and Hsp90β) interact with more clients than the ER resident GRP94 and mitochondrial localized TRAP1. For instance, the interaction list of HSP90 includes kinases such as Akt2, CDKs, PKC, many MAP kinases as well as transcription factors like steroid receptors, BCL-6, CAR, p53, Oct4. A valuable detailed information about the cytoplasmic HSP90 protein clients and co-chaperones is available on the web site sustained by Dr. Didier Picard at http://www.picard.ch/downloads/Hsp90interactors.pdf. 

### 4.2. GRP94 and TRAP1

For these two HSP90 isoforms, the protein client list is relatively short but subject to increase. Sample clients are summarized in the following Table 3.

## 5. Mode of Action

### 5.1. ATPase Activity

**Figure 2:**
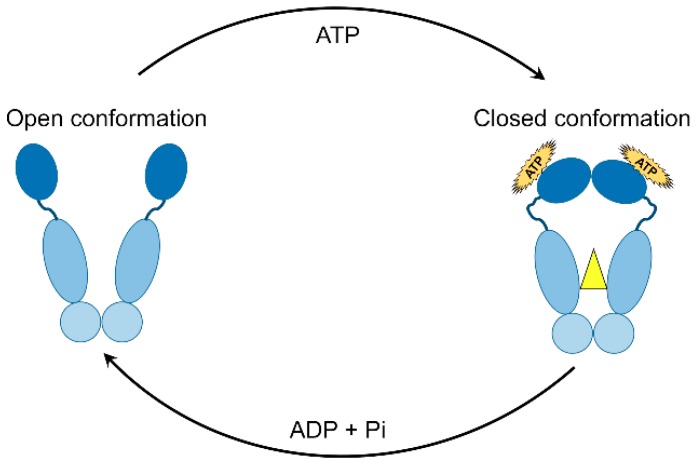
Schematic representation of the ATPase cycle of HSP90. The open and closed conformations of HSP90 are depicted.

The central dogma in the HSP90 mechanism of action is its ATPase activity and cycling between the closed and open states [94]. Although located in different compartments inside the cell, all HSP90 isoforms including cytoplasmic, ER, and mitochondrial homologues that act in a similar way in terms of conformational alterations following nucleotide binding [95]. As described earlier, HSP90 exists as a flexible homo-dimer whose monomers consist of three structural domains; NTD, MD, and CTD. Binding of ATP occurs in the ATP binding cleft in the NTD of HSP90 resulting in series of conformational events. An interesting event includes a short piece of the N-domain (ATP-lid), which trans-locates over the binding pocket and attaches to the corresponding N-domain of the other homo-dimer resulting ultimately in a twisted and compacted dimer [96]. As a result, the N- and M-domains get closer by 40 Å leading to finalizing the “split ATPase” site. After ATP hydrolysis, the N domains of the HSP90 homo-dimers dissociate with the release of ADP and Pi while the HSP90 returns to its original open conformation (Figure 2) [13]. Interestingly, ATP hydrolysis in humans occurs at a rate of 10–20-fold slower than in yeast [30,97], suggesting a complex conformational rearrangement of HSP90 in higher eukaryotes [95]. Critically, the well-known anticancer drugs geldanamycin and radicicol bind specifically to HSP90 in a fashion similar to how ATP does, thus serving as potent inhibitors of HSP90 ATPase activity [98,99].

### 5.2. Chaperone Cycle

**Figure 3:**
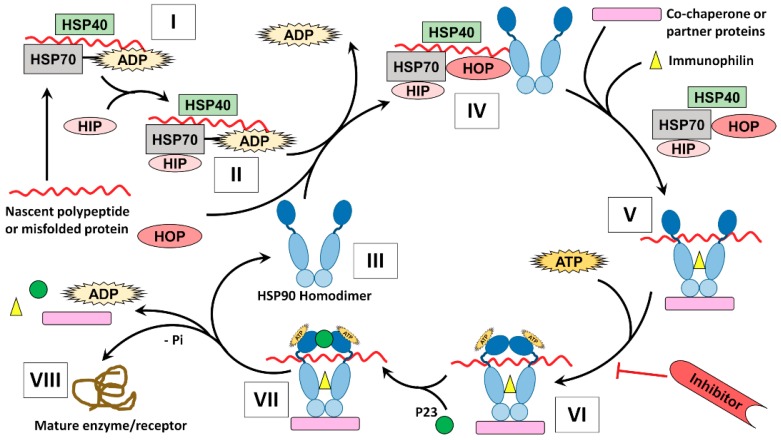
The mechanism of protein folding by HSP90 [44]. (**I**) The client protein either mis-folded protein or nascent polypeptide is bound by Hsp70/Hsp40/ADP complex to be protected from potential aggregation [102]; (**II**) The protein complex formed is further stabilized by Hsp70-interacting protein (HIP) or Bcl2 which support the change of ADP to ATP [103]; (**III**) HSP90 binds the client protein harboured by the Hsp70/Hsp40 protein complex to start its action; (**IV**) The interaction between HSP90 and HSP70 is facilitated by the adaptor protein, HOP/Sti1 (Hsp90–Hsp70 organizing protein) [104]. Importantly, additional co-chaperone known as Cdc37 (cell-division-cycle 37 homologue) or p50 is needed for loading client kinases onto HSP90 [105]; (**V**) As the client protein is loaded, other co-chaperones, immunophilins like (FKBP51, FKBP52) are added to the HSP90 homodimer forming an activated heteroprotein complex while the HSP70, HIP and HOP are released [106]; (**VI**) Binding of ATP to the NTD of HSP90 in the heteroprotein complex switches the HSP90 from the “open state” to the “closed state” [107]. Various inhibitors of HSP90 can act at this stage by competing with ATP for the NTD binding site consequently resulting in protein degradation by proteasome [101]; (**VII**) If no inhibitors interfere with the folding cycle, association of other co-chaperones occurs including p23 and Aha1 (activator of Hsp90 ATPase homologue 1); (**VIII**) Binding of Aha1, not shown in the diagram, to the MD of HSP90 induces ATP hydrolysis and supports folding of the bound client, as well as enhances the release of immunophilins and co-chaperones [96].

The members of HSP90 family perform their folding function of client proteins through a complicated process known as the HSP90 chaperone cycle. Previous reviews demonstrated how HSP90 mediates the folding of its client proteins, however the complete understanding of the protein folding cycle is yet unreached [13,100,101]. In fact, the HSP90 chaperone machinery requires cooperation of different molecules including co-chaperones, partner proteins, and immunophilins, which act in precise and dynamic manner helping efficient protein folding by HSP90 (Figure 3). 

## 6. Roles of HSP90 Isoforms in Cellular Physiology

### 6.1. Cytosolic HSP90

#### 6.1.1. Chaperone Function

Several previous reports demonstrated the role of HSP90 in protein folding and preventing protein aggregation [108,109,110]. Although HSP90 requires ATP for its action, purified HSP90 could stabilize heat-denatured proteins, including citrate synthase, rhodanase, and protein kinase CK-II [111,112]. In addition, HSP90 prevented further aggregation of mild aggregates of CK-II resulting from low salt exposure [108,113]. Furthermore, experiments involving heat-denatured luciferase [114] and guanidinium.HCl-denatured β-galactosidase [114] showed that HSP90 confers folding competence to these molecules and facilitates their refolding by other chaperones [114]. In addition to its ATP-dependent activity, the ATP-devoid chaperone activity of HSP90 discriminates it from other HSPs like HSP60 and HSP70 [115] and provides complexity to its mode of action [13,116]. Noteworthy, HSP90 acts in cooperation with other chaperones, including HSP70 and HSP40, as well as co-chapeones, like TPR-containing co-chaperones, to refold many denatured proteins, such as steroid receptors and protein kinases [63].

#### 6.1.2. Cell Signaling

HSP90 protein clients are associated with several signaling pathways like steroid hormone receptors and protein kinases (the link to the current client list is provided above). Similar to steroid receptors, HSP90 appeared to be essential in the maturation of the aryl-hydrocarbon (dioxin) receptor [117]. Other reports demonstrated that decreased expression of HSP90 deactivated retinoid receptors and hindered the development of high-affinity retinoid acid binding [118]. Likewise, HSP90 formed complexes with a large number of protein kinases where the first-described protein kinase in complex with HSP90 was the v-Src tyrosine kinase [119,120]. Binding of v-Src to HSP90 causes hypophosphorylation and abolishes protein kinase activity [18]. Raf kinase also forms a complex with HSP90 which facilitates its membrane association [121]. HSP90 has been shown to protect kinases from the action of phosphatases [122]. Interestingly, premature dissociation of HSP90 complexes, including Src- and Raf- hsp90 complexes, could be achieved by the hsp90-specific drugs geldanamycin and radicicol [123]. Dissociation of such kinases ultimately leads to enhanced proteasomal degradation [124]. Other kinases such as the tetratricopeptide domain-containing immunophilin (PP-5) and transcription factors have been reported to be a part of the HSP90 complex. Therefore, it is not surprising that HSP90 is designated as a key chaperone implicated in cell signaling.

#### 6.1.3. Cytoskeleton

Due to its abundance and adhesion properties, cytosolic HSP90 was named as “molecular glue” [18]. HSP90 was found to crosslink actin filaments in vitro [25]. However, HSP90 was not co-localized with actin in human fibroblasts or human endometrial adenocarcinoma cells. Many early studies suggested a dynamic transient interaction of HSP90 with almost all cytoplasmic filamentous structures highlighting its role in maintaining the cyto-architecture [18]. Further studies showed that binding of HSP90 to actin filaments favors an ATP-lacking environment which mostly occurs in severe stress conditions [125,126]. Additionally, HSP90 appeared to modulate actin filament bundling behavior [127]. In addition to actin, HSP90 has been reported to interact with tubulin and protect it from heat denaturation [128], microtubules [129], and other non-microtubular and non-microfilamental structures of the cytoplasm. Furthermore, HSP90α has been shown to protect myosin from heat stress [130]. Therefore, HSP90 is involved in the arrangement and maintenance of almost the whole cytoskeleton.

#### 6.1.4. Nuclear Functions

Under normal conditions, about 5–10% of total HSP90 exist in the nucleus of the cell. This nuclear localization increases upon heat stress [18,131]. HSP90 is associated with nucleoli and perichromatin ribonucleoprotein fibrils [132]. In fact, HSP90 contains a bipartite nuclear localization sequence in the middle, highly-charged region of the protein. Additionally, nuclear import of HSP90 is modulated by FKBP52, steroid receptors, and kinases [27,133]. Furthermore, the sequence of HSP90 contains other sequences homologous to the recognized traditional or alternative nuclear import and export signals [18]. Since it served to transport steroid receptors and kinases to the nucleus, it has been suggested that HSP90 binds NLS [134]. Importantly, HSP90 binds to DNA, RNA, and histones where it modulates the structure of DNA and contributes to RNA synthesis and processing [18]. In addition to histones, HSP90 interacts and forms complexes with many transcription factors, including zinc finger proteins, helix-loop-helix proteins, MyoD1, E12, hypoxia-inducible factor 1α, and heat-shock factor 1 [18].

#### 6.1.5. Cell Cycle and Cellular Differentiation

HSP90 and the co-chaperone CDC37/p50 fold cell cycle-related protein kinases including cyclin-dependent kinase CDK4, cyclin-dependent kinase CDK11p110 [135] and the cyclin-dependent kinase regulator Wee1 [136]. Interestingly, the expression profile of HSP90 isoforms differs according to the stages of the cell cycle [137]. Transcription of HSP90α is stimulated at the G1/S transition of chicken hepatoma cells [138]. In fibroblasts, the ATP concentration has been shown to rise two-fold during mitosis [139]. Variations in ATP levels during cell cycle stages dramatically affect the functions of HSP90 isoforms. In addition to cell cycle changes, cell differentiation also caused alterations of HSP90, as well as GRP94 expression. Differentiation of embryonal carcinoma cells resulted in upregulation of HSP90β [140]. Interestingly, the expression level of HSP90α and HSP90β is reduced during osteoblast and HL-60 cell differentiation [141] suggesting that the expression pattern of HSP90 during differentiation is cell-type dependent [18]. Additionally, genetic mutation of HSP90β led to diminished hepatocyte differentiation from induced pluripotent stem cells [142]. HSP90 was found to stabilize the peroxisome proliferator-activated receptor-γ (PPARγ) thus, regulating adipose tissue differentiation and function [143]. Another interesting point, the expression profile of the cytoplasmic isoforms Hsp90α and HSP90β change during the differentiation of myoblasts into myotubes where HSP90β dominates and interacts with the muscle-specific HSP90 co-chaperone Aarsd1L [144].

#### 6.1.6. Extracellular HSP90 or Secreted HSP90

Generally, initial reports concerning the idea of HSP secretion faced difficulties in convincing the scientific community about the fact that HSPs are released or secreted from intact cells [145]. Later, HSP70 was proven to be secreted from cultured rat embryo cells [146]. Further studies confirmed the secretion of many classes of HSPs, including small HSPs and HSP90, from different cells where they are suggested to perform specific immunological functions [147]. HSP90α has been secreted from cells following many stresses such as reactive oxygen species, heat, hypoxia, irradiation, and tissue injury release cytokines [15]. There has been a strong notion that normal cells do not secrete HSP90 provided being elicited by environmental influences [148]. Further studies suggested that the secretion of HSP90α occurs as a result of TGFα induction [149] and is regulated by the hypoxia-inducible factor-1alpha (HIF-1α) [150,151]. eHsp90α has been shown to enhance cell motility and support wound healing [148]. Interestingly, it was found that eHSP90α acts in a way different from its cytosolic homologues as the ATPase activity was not required for its function in cell motility [15]. For that, eHSP90 function was dependent on a fragment (F-5) including residues 236–350 and located between the linker region and the middle domain [15,152]. 

### 6.2. GRP94

#### 6.2.1. Protein Folding

As a molecular chaperone, the main task of GRP94 is to promote the folding and assembly of secretory and membrane proteins [49]. An important characteristic that distinguishes GRP94 from other ER chaperones is its functional selectivity, which is reflected by its relatively short client protein list. In agreement with the selectivity hypothesis, GRP94 is not essential for folding or trafficking of various plasma membrane receptors and secretory molecules including transferrin receptor and MHC class I [49,90]. Moreover, in contrast to BiP, which has been shown to bind both V and C domains, GRP94 binds specifically to the light chain through Ig-fold variable domains. Furthermore, secretion of light chains was inhibited in GRP94 deficient B-cells [153]. Bile salt-dependent lipase (BSDL), also known as cholesterol esterase, represent another example of GRP94 client proteins forming complex to be secreted from pancreatic cells [154,155]. Known client proteins of GRP94 include Toll-like receptors and integrins, which require the ER chaperone for efficient trafficking and cell surface expression [156,157]. Interestingly, gp93 the drosophila homologue of GRP94 could help surface expression of many integrins and TLRs in mammalian cells lacking GRP94 [157]. Also, GRP94 interact with the platelet glycoprotein Ib-IX-V complex, a receptor for Willebrand factor whose absence results in defective platelets function. The interaction with GRP94 guarantees good surface expression and improves the ER-associated degradation (ERAD) of the complex [158]. Other clients include thyroglobulin, insulin-like growth factors, and others that have been listed in Table 3.

#### 6.2.2. Calcium Homeostasis

GRP94 is a calcium binding protein which has low affinity, high capacity binding to calcium [46,159]. Together with other ER chaperones, it mediates calcium homeostasis in the ER and the cell. Although important, calcium binding by GRP94 was found not essential for life. Skeletal muscle cells which lacked GRP94 did not show clear defect in calcium homeostasis suggesting another compensating mechanism for calcium by calsequenstrin-1 [160].

#### 6.2.3. ER Quality Control

To produce correctly folded secretory and membrane proteins, there is a network of chaperones inside the ER which control and maintain this process. GRP94 acts as a major checkpoint in this network. The function of GRP94 in the quality control of the ER can be summarized in terms of (1) chaperoning protein folding intermediates; (2) interaction with other components of the ER protein folding machinery; (3) calcium storage; and (4) helping targeting of mis-folded proteins to ERAD [161].

#### 6.2.4. ER Stress

GRP94 and BiP (GRP78) are considered the hallmarks of the unfolded protein response (UPR). Both chaperones and other ER chaperons have analogous promoters, therefore, usually induced concomitantly under physiological as well as pathological conditions [49]. Overexpression of GRP94 have been reported in physiological events like differentiation of hepatic cells, acinar cells, and B cells [49,162]. Enhanced folding and promotion of IGF-1 and IGF-II by GRP94 leads to increased hormone output under stress and boosting IGF pathway. These consequences lead to improving the UPR, increasing the folding capacity of the ER, suppression of apoptosis and alleviating metabolic stresses [49].

#### 6.2.5. Extracellular GRP94

Earlier investigations reported that GRP94 can be released outside the cells where its secretion was associated with severe tissue damage and necrotic cell death [163,164]. These studies suggested that necrotic, rather than apoptotic, cells produce extracellular forms of the ER chaperone [163]. GRP94 has been known to chaperone intracellular peptides as well as tumour-specific peptide and present them on the MHC class I molecules of antigen presenting cells (APCs) in a process called “cross-presentation” [165,166,167]. Moreover, although mainly localized in the ER, GRP94 was expressed on the cell surface of tumour cells [168]. Evdokimovskaya and his colleagues reported the release of GRP94 from BHK cells under normal culture conditions [165]. Moreover, we reported the secretion of GRP94 from other cell lines including COS-1 and CHO-K1 cells as well [47]. In fact, other ER chaperones like GRP78 and calreteculin have been reported to be released to the extracellular space where they are suggested to display immunogenic and inflammatory related roles [147,169]. These data strongly point to the possibility that released forms of GRP94 can perform immunogenic functions even under non-pathologic conditions. However, this notion requires further investigation [47]. 

### 6.3. TRAP1

#### 6.3.1. Role of TRAP1 in Mitochondria

The major function of TRAP1 is to maintain mitochondrial integrity and protect against mitochondrial apoptosis [55]. Oxidative stress resulting from increased production of oxygen free radicals or reactive oxygen species (ROS) is the main cause of mitochondrial dysfunction and cell death. Various diseases such as neurodegenerative, cardiac, neoplastic maladies and stroke [170] are potential consequences of excessive ROS production. It has been shown that TRAP1 can protect against cell death induced by over production of ROS [171,172]. Over-expressed TRAP1 could reduce ROS accumulation while its knock-down increased ROS formation [173,174]. Furthermore, TRAP1 counteracts protein aggregation inside the mitochondria and support protein folding [175] leading to healthy, intact mitochondria. 

#### 6.3.2. How Does TRAP1 Protect Against Oxidative Stress?

Accumulated ROS causes opening the permeability transition (PT) pore of the inner mitochondrial membrane leading to disturbed membrane potential [176]. As a result, the mitochondria are swollen and the outer membrane can be ruptured resulting in cell mortality [177]. Studies on PT pore showed that the voltage-dependent anion channel (VDAC), located in the outer mitochondrial membrane, and the adenine nucleotide translator (ANT), located in the inner mitochondrial membrane, are not the lone PT pore components [178,179,180]. Critically, the mitochondrial matrix protein, cyclophilin D (Cyp-D), was found to play a key role in the pore formation of the mitochondrial inner membrane [181]. Being a peptidyl-prolyl cis-trans isomerase located in the mitochondrial matrix, Cyp-D supports protein folding of the PT pore proteins inside the mitochondria. In addition, increased ROS, calcium, and diminished ATP following stress conditions cause activation of Cyp-D which, in turn, modulate the conformation of PT pore proteins and consequently generate non-selective pores through the membrane [177,182,183]. TRAP1 is a major interacting protein with Cyp-D, hence, greatly influences its function. For instance, TRAP1 can hinder the Cyp-D pore opening activity in various cell types including tumour cells allowing cell survival [184,185]. Although the ATPase activity of TRAP1 has been thought to be essential in its inhibiting ability of Cyp-D, treatment of cells with TRAP1 inhibitors did not affect its function regarding Cyp-D inhibition [185]. These findings indicate that the chaperone activity rather than the interaction issue is the mainstay in Cyp-D inhibition by TRAP1 [60].

#### 6.3.3. Extra-Mitochondrial Roles

A cohort of the mitochondrial TRAP1 molecules can be trans-located outside the mitochondria and bind extra-mitochondrial proteins. TRAP1 has been reported to interact with TNF receptor [56] retinoblastoma protein [74], and tumour suppressor EXT proteins [76]. Additionally, during mitosis, TRAP1 interacts with the retinoblastoma protein to help its folding [74]. Similar to TRAP1, the tendency to perform extra-mitochondrial tasks is a common feature of other mitochondrial chaperones, like HSP60 and mortalin, especially in cancer cells [186]. Although the role of TRAP1 in maintaining mitochondrial integrity, defending against oxidative stress, and inhibiting cell death have been addressed in many reports, how it is exported to the cytoplasm and what it exactly does there is still beyond our knowledge [60].

## 7. Role of HSP90 Isoforms in Pathological Conditions

Disease, in general, induces a kind of stress on the cellular machinery. Therefore, variant types of HSPs including HSP90 are involved in diverse pathological conditions like cancer, neurodegenerative diseases, infectious diseases, and others. Here, we discuss the major common diseases that have been linked to members of HSP90 family.

### 7.1. Cytosolic HSP90

#### 7.1.1. Cancer

Normal mammalian cells are equipped with defined molecular machinery sufficient to regulate their proliferation, growth, differentiation, and death. On the other hand, cancer cells are characterized by persistent uncontrolled proliferation and lacks the native regulatory mechanisms responsible for normal cell proliferation and homeostasis [187]. Several cancer proteins depend on HSP90 machinery and chaperones for their folding and maturation [188]. For instance; signaling kinases, steroid hormone receptors and transcription factors, which are highly required in cancer development and progression, represent a group of HSP90 client proteins (Table 4). Therefore, pronounced expression of HSP90 has been detected in almost all classes of cancers. A new theory named “addiction to chaperones” has been adapted to explain the great demand for HSPs including HSP90 during cancer growth [22]. According to this hypothesis, overproduction of mutant proteins in cancerous cells act as a fuel for HSPs induction, increased translational processes, and expression of proteins leading to transformation [22].

##### HSP90 as a Target for Anti-Cancer Drugs

Due to the higher sensitivity of cancer cells to HSP90 inhibitors than healthy cells, the cytosolic molecular chaperone HSP90 has attracted a great deal of interest in the therapeutic field of cancer. Natural products like geldanamycin (GA) that was derived from *Streptomyces hygroscopicus* and the antibiotic radicicol (RD), isolated from *Monosporium bonorden* exhibited prominent suppression of tumor proliferation and growth [189,190]. Having similar structures to ATP, these HSP90 inhibitors bind the ATP binding pocket of the NTD within the HSP90 structure, thus interfering with ATP binding and hydrolysis, consequently leading to the depletion of HSP90 oncogenic clients [98,191]. Although the anti-cancer efficacy of GA has been tested in many in vivo and in vitro studies, it has not yet been approved for clinical uses due to its structural instability and hepatotoxicity. However, GA is still commonly used for in vitro studies concerning HSP90 inhibition [192,193]. Similarly, RD usage was limited due to its instability as well [194]. Further chemical modification of GA specifically by substituting its C_17_ with an allylamino group, producing 17-AAG (17-allyl-17-demethoxygeldanamycin) greatly enhanced its toxicity profile [195]. Therefore, 17-AAG have been used in phase I/II clinical trials [190,196]. Other strategies suggest a combination therapies including 17-AAG and other chemotherapeutic drugs or radiotherapy as a successful approach for cancer therapy [15]. Recently, many other derivatives have been developed from GA and RD, including second-generation HSP90 inhibitors, along with other drugs, which target the middle and C-terminal domain of HSP90 (see [190,197] for reviews).

#### 7.1.2. Neurodegenerative Diseases

HSP90 plays an additional role in protecting neuronal proteins with aberrant aggregation tendency from accumulation and forming toxic aggregates [8]. HSP90 counteracts protein aggregation accompanying neurodegenerative diseases like Alzheimer disease and Parkinson disease [63]. Intriguingly, in Alzheimer disease HSP90 appeared to promote the hyper phosphorylation of tau protein (tau-p) via activation of kinases leading to the formation of neurofibrillary tangles [63,198]. Targeting HSP90 by inhibitors led to reduction in p-tau levels through a mechanism mediated by the tau ubiquitin ligase; carboxy terminus of Hsp70–interacting protein (CHIP) [198]. In Parkinson disease, in vitro experiments revealed that HSP90, as well as HSP70/40 could suppress Aβ amyloid self-assembly suggesting that induction of chaperones’ overexpression by pharmacological substances has potential therapeutic value for neurodegenerative diseases [110]. In agreement with this hypothesis, overexpression of HSP70, following treatment with drugs inducing its expression as geldanamycin, could reduce α-synuclein induced neurotoxicity in human H4 neuroglioma cells, as well as a fly model of Parkinson disease [199,200,201]. 

#### 7.1.3. Infectious Diseases

##### Viral Diseases

Increasing reports suggest the involvement of HSP90 in viral protein homeostasis [9]. Numerous viral proteins have been shown to utilize HSP90 for folding, assembly and maturation. Viral polymerases represent the major subset of viral proteins requiring HSP90 machinery for their processing. For instance, reverse transcriptase (RT) of duck hepatitis B virus (DHBV) needs HSP90 together with other chaperones, including HSP70/HSP40, and a co-chaperone, like HOP and p23, as substrate release factors and supporting incorporation of the pgRNA into nucleocapsids [202,203,204]. Additionally, the genome replication of influenza virus A needs HSP90 for its replication [9]. A detailed list of viral proteins requiring HSP90 has been reported previously [9]. It seems that the demand for HSP90 chaperone in viral infections comes from the increased translational rates of viral proteins, high incidence of mutations, as well as high conformational and proteolytic events. These stressful processes necessitate the presence of HSP90 as a buffering system [63]. 

##### Parasitic Diseases

Interestingly, the malarial parasite *Plasmodium falciparum* requires the HSP90 machinery in the human host to withstand the surrounding environmental stresses including pH and periodic elevated temperature fluctuations in the patient [205]. Similarly, *Leishmania donovani*, the causative agent of leshmaniasis, requires HSP90 during its life cycle and pathogenicity [206]. Therefore, it is not surprising that Hsp90 forms about 3% of total protein content in the promastigote stage of *L. donovani* [207]. Moreover, there are 17 homologues of HSP90 genes in *Leishmania major* presumably functioning in its pathogenesis [15,208]. In other nematodes, HSP90 has been reported to affect the morphological features by canalizing the mouth-form plasticity in *Pristionchus pacificus* [209]. 

#### 7.1.4. Other Diseases

HSP90 is implicated in the pathogenesis of other serious diseases, like cystic fibrosis (CFTR), atherosclerosis, and diabetes. The deletion mutation (ΔF508), of CFTR, seriously affect the protein folding and causes its degradation [63]. HSP90 was found to interact with the wild-type, as well as mutant, CFTR. As a part of the HSP90-protein complex, AHA1 appeared to play a key function in stabilizing the mutant CFTR within the HSP90 chaperone complex. Knockdown of AHA1 leads to a high number of mutant CFTR channels transferred to the plasma membrane, suggesting that the AHA1 co-chaperone mediates processing and degradation of mutant CFTR by blocking its escape from the chaperone cycle [63,210]. 

In patients with atherosclerosis, expression of HSP90 is increased in atherosclerotic plaques and has been associated with plaque instability [211]. Interestingly, HSP90-inhibiting drugs could diminish plaque formation by suppressing the migration and proliferation of vascular smooth muscle cells (VSMCs) [211]. Other studies reported that the high HSP90 expression is not restricted to the atherosclerotic plaques. The serum of atherosclerotic patients contained elevated HSP90 levels presumably inducing immune response, thus, HSP90 have been considered as a possible target auto-antigen in the pathogenesis of carotid atherosclerosis [212]. 

Recent studies reported elevated HSP90 levels in newly-diagnosed type I diabetic patients suggesting HSP90 as a potential biomarker for this serious disease [213]. Additionally, experiments in a mouse model for diabetes-accelerated atherosclerosis showed that inhibition of HSP90 was found to reduce both inflammation and oxidative stress in addition to limiting vascular and renal damage [214].

### 7.2. GRP94

#### 7.2.1. Cancer

As a molecular chaperone implicated in the quality control of the ER, it is not surprising that GRP94 chaperones several proteins essential in carcinogenesis [215], including TLRs (with the exception of TLR3) [156], Wnt co-receptor LRP6 [216], GARP [217], GPIb [158], and Insulin-like growth factor [218], as well as most of the α and β integrin subunits [219,220]. GRP94 has been known as an active tumor rejection antigen that can immunize recipients against tumor transplants [221]. Complexes of GRP94 and its chaperoned tumor peptides rather than GRP94 alone seemed to be responsible for GRP94 immunogenicity. Overexpression of GRP94 has been reported in multiple types of neoplasms where high GRP94 expression was clinically indicative for advanced cancer stage and poor prognosis [215], like head and neck cancer [222], gallbladder cancer [223], and breast cancer [224]. Other studies showed that overexpressed GRP94 encouraged the growth and metastasis of certain cancers, including hepatocellular carcinoma [225], multiple myeloma [226], ovarian cancer, and inflammatory colon carcinomas [227]. Localization studies revealed that GRP94 favors cell surface expression in cancerous cells suggesting excess immunogenic roles [228,229]. These immunological functions include increased antigen presentation and enhanced T-cell activation [229]. Therefore, it can be concluded that GRP94 exhibits double roles in carcinogenesis; (1) supporting cancer progression, in terms of assisting the folding of cancer related proteins; and (2) induction of immune response against cancer through cross-presentation of tumor antigenic peptides and T-cell activation [215].

##### GRP94 as a Target for Anti-Cancer Drugs

It has been shown that cancerous cells exhibit high surface expression of GRP94, although it is ordinarily localized in the ER [221]. Other studies demonstrated that increased cell surface expression of GRP94 is associated with tumor immunogenicity [228]. Further studies proved that GRP94 could present antigenic peptides which have anti-tumor activity [230] highlighting the critical role of GRP94 in induction of immune response against tumors [215]. Theses previous studies were the basis for GRP94-based antitumor therapies. GRP94-based antitumor vaccines could protect against methylcholanthrene-induced fibrosarcomas (Meth A) tumors [231]. Specific monoclonal antibodies against GRP94 were used as powerful tools to inhibit cancer growth and development in tumor tissues. For instance, the monoclonal antibody (W9 mAb) was recently used and precisely targets the extracellular epitope of gp96 which is barely found in normal cells [232]. Another study used GRP94 or gp96 monoclonal antibody (gp96 mAb) to interrupt the HER2 dimerization and phosphorylation in breast cancer, which lead ultimately to inhibiting their growth and proliferation both in vitro and in vivo [215].

Chemicals targeting GRP94 have also been used as promising anti-cancer remedies. The main concern in this strategy was the pan- inhibition of other HSP90 family members in normal cells as well which may lead to disruption of vital biological process relying on them [233]. This underlines the need for developing more specific and selective anti-cancer therapies. Documented examples of GRP94 selective drugs include 5′-*N*-ethylcarboxamidoadenosine (NECA), Compound 2, and Radamide [234,235]. Other strategies developed selective GRP94 compounds based on conformational investigations and screening of purine-scaffold compounds. These studies produced gp96 inhibitory ligands as PU-H54, PU-WSI3, and PU-H39, which had distinct structural features in the ATP-binding pocket [236]. In summary, there are promising beneficial clinical impact for developing gp96-selective inhibitors rather than using drugs with pan-inhibition effect on the HSP90 machinery [215]. 

#### 7.2.2. Neurodegenerative Diseases

Among the major consequences of neurodegenerative diseases is the accumulation and aggregation of mis-folded proteins within almost all cellular compartments. Cellular responses including ER stress, unfolded protein response (UPR) and over expression of HSPs serve as protective mechanisms to counteract these protein aggregations [237,238]. The ER chaperone GRP94 is a key player in the quality control of the ER helping refolding or disposal of mis-folded and aggregated proteins [161]. Increased secretion of GRP94 and lower intracellular levels have been associated with amyloid-β (Aβ) peptide-induced neurotoxicity in rat neuronal-like PC12 cells [239]. Recently, a vaccine composed of combination of α-synuclein (α-Syn) and Grp94 chaperone could provide a marked long-lasting immune profile in the peripheral system, which has an influence on the CNS by inhibiting chronic microglial activation in chronic mouse 1-methyl-4-phenyl-1,2,3,6,-tetrahydropyridine (MPTP)-model of Parkinson’s disease (PD) [240].

#### 7.2.3. Infectious Diseases

##### Viral Diseases

GRP94 or GP96 has been reported to be involved in viral infections. GRP94 interacts with and supports protein folding and export of Toll like receptors (TLRs) [241]. Other studies confirmed the role of TLRs as key sensors of innate immunity to viruses by identifying their components like nucleic acids and envelope glycoproteins [242]. Following viral infections, activated TLRs, trigger several signaling events resulting in production of type I interferons, inflammatory cytokines, and chemokines ultimately leading to eliminating an invading pathogen [243]. In addition to its role in the functionality of TLRs, GRP94 was found to be essential in Vesicular stomatitis virus infection [244]. Overexpression of GRP94 has been reported in Rota virus-infected cells [245]. Patients with Hepatitis B virus (HBV) displayed high expression levels of GRP94 which has been associated with poor prognosis [246,247]. GRP94 appeared to be a key regulator in the stabilization and regulation of HBV RNA polymerase [248,249]. Studies with Japanese encephalitis virus revealed that unlike HSP90 α which was retained inside the nucleus, GRP94 was released into the secretion medium upon JEV infection [250].

##### Parasitic Diseases

A single copy of GRP94 has been characterized in *Leshmania major* where it has been linked to its virulence [208]. In addition, three genes of GRP94 has been found in the Chagas’ disease causing parasite, *Trypanosoma cruzi*. Notably, the HSP90 inhibitor geldanamycine hindered the parasite development and seemed to interfere with proteins involved in the epimastigote differentiation [15,208]. Importantly, the secreted forms of GRP94 which chaperones *plasmodium falciparum* (Pf) AMA1 and PfCSP sporozoite proteins have been recently exploited in developing a novel vaccine against malaria [251].

#### 7.2.4. Other Diseases

The involvement of GRP94 in several diseases comes from its important role in the ER quality control through folding of newly synthesized proteins and proteostasis [49,161]. For instance, mutation-causing diseases or diseases causing ER stress, including atherosclerosis, obesity, and diabetes, upregulate GRP94 and other members of the ER chaperone network to deal with the resultant ER stress. In agreement with these facts, GRP94 has been found critical for regulation and development of pancreatic β-Cells providing new insights for preservation enhancement of β-cell function. [252].

### 7.3. TRAP1

#### 7.3.1. Cancer

TRAP1 participates in the carcinogenesis of many tumors [253]; (1) it interferes with aerobic glycolysis by inhibiting succinate dehydrogenase; (2) supports cell survival through hindering ROS accumulation and counteracting toxic cancer drugs and oxidants; and (3) maintains protein homeostasis by regulation of de novo protein synthesis and protein degradation. Additionally, in cancerous tissues, contrasting reports about TRAP1 expression patterns have evolved [254]. High expression of TRAP1 has been reported in colon cancer [255,256], breast cancer [257,258], prostatic cancer [259,260], lung cancers [261], and glioblastoma [262]. On the other hand, reduced expression has been shown in particular tumors, such as ovarian [263,264], bladder, and renal cancers [265]. Interestingly, high TRAP1 expression has been linked to drug resistance while its downregulation has been associated with poor prognosis. The controversial expression data of TRAP1 in cancer suggested a potential dual role as an oncogene or onco-suppressor according to cancer type [254].

#### 7.3.2. Neurodegenerative Diseases

In addition to TRAP1 function in keeping mitochondrial integrity under normal physiology, it has been shown to contribute in correcting against mitophagy and mitochondrial degeneration in many neurodegenerative diseases. As a substrate for Parkinson’s disease kinase (PINK1), phosphorylated TRAP1 sustains healthy mitochondria by suppressing cytochrome C release from mitochondria. Impaired phosphorylation of TRAP1, due to mutated PINK1, leads to Parkinson’s disease [68,253]. Moreover, neuronal cancer cell lines that lacked TRAP1 such as neuroblastoma and glioma showed irregular mitochondrial morphology with diminished levels of dynamin-related protein 1 (Drp1) and mitochondrial fission factor (Mff) [266]. In fact, *Drosophila* has been extensively used as a model for PD. Interestingly, human TRAP1 could rescue PINK1 loss-of-function phenotypes in *Drosophila* and act against mitochondrial fragmentation and dysfunction in human neuronal SH-SY5Y cells [267]. In other studies, Drosophila TRAP1 null mutants revealed impaired mitochondrial function and hypersensitivity to stress suggesting a down-stream role of TRAP1 following PINK1 in mitochondrial metabolic pathways [268]. In vitro experiments revealed that A53T mutated α-Synuclein disrupts mitochondrial functionality in terms of insufficient ATP synthesis, mitochondrial fragmentation and elevated oxidative stress. However, overexpression of human TRAP1 was able to relieve these deleterious effects [269]. Recent studies revealed that TRAP1 is a novel interactor of protease HTRA2 and mutations in both proteins cause PD [270]. The TRAP1 mutation R47X caused increased mitochondrial respiration and ATP production [271]. These consequences were similar to TRAP1 silencing in ovarian cancer which led to resistance to chemotherapy due to increased oxidative phosphorylation [264]. Interestingly, the diabetes drug Metformin could alleviate the TRAP1 mutation associated disturbances of energy metabolism in PD through mild inhibition of mitochondrial respiration [271].

#### 7.3.3. Kidney Diseases

TRAP1 have been reported to be involved in renal diseases. Overexpression of TRAP1 in glomerulonephritis was accompanied by DNaseI shutdown, which happens in end-stage disease, suggesting transcriptional interference between TRAP1 and DNaseI genes [272]. Moreover, using deep sequencing, revealed common recessive mutations of TRAP1 among patients with congenital anomalies of the kidney and urinary tract (CAKUT) [253,273].

## 8. Conclusions and Perspectives

The 90 kDa HSPs constitute a class of highly conserved proteins implicated in a plethora of physiological, as well as pathological, processes. Due to their abundance and distinct cellular distribution, the function of HSP90 members attracted researchers to extensively explore their complexity. Although we present here the most significant knowledge about HSP90 family members, it only represents a fraction of the HSP90 literature. In this review we shed light on the major diverse functions of HSP90 family members in terms of structure, regulation, and function in health and disease conditions. The use of HSP90 inhibitors is of critical clinical importance especially in cancer and neurodegenerative diseases. However, the challenge remains to identify and establish the most effective and least toxic drug.

## Figures and Tables

**Table 1:** Different members of human HSPC (HSP90) family [5].

Gene Name	Protein Name	Alternative Name	Human Gene ID
*HSPC1*	HSPC1	HSP90AA1; HSPN; LAP2; HSP86; HSPC1; HSPCA; HSP89; HSP90; HSP90A; HSP90N; HSPCAL1; HSPCAL4; FLJ31884	3320
*HSPC2*	HSPC2	HSP90AA2; HSPCA; HSPCAL3; HSP90ALPHA	3324
*HSPC3*	HSPC3	HSP90AB1; HSPC2; HSPCB; D6S182; HSP90B; FLJ26984; HSP90-BETA	3326
*HSPC4*	HSPC4	HSP90B1; ECGP; GP96; TRA1; GRP94; endoplasmin	7184
*HSPC5*	HSPC5	TRAP1; HSP75; HSP90L	10131

**Table 2:** Contributing co-chaperons and proteins in the HSP90 chaperone folding cycle [44,69].

Co-Chaperone or Protein Partner	Role in Carcinogenesis
Aha1	Stimulates ATPase activity
Cdc37	Mediates activation of protein kinase substrates
CHIP	Involved in degradation of unfolded client proteins
Cyclophilin-40	Peptidyl propyl isomerase
FKBP51 and 52	Peptidyl propyl isomerase
HOP	Mediates interaction between Hsp90 and Hsp70
Hsp40	Stabilizes and delivers client proteins to Hsp90 complex
Hsp70	Stabilizes and delivers client proteins to Hsp90
p23	Stabilizes closed, clamped substrate bound
HIP	Inhibits ATPase activity of Hsp70
PP5	Protein phosphatase 5
Tom70	Facilitates translocation of pre-proteins into mitochondrial matrix
Sgt1	Client adaptor, involved in client recruitment
WISp39	Regulates p21 stability

**Table 3:** Protein clients of GERP94 and TRAP1.

GRP94 Client Protein	Citation	TRAP1 Client Protein	Citation
MHC class II	[72]	TNF receptor	[56]
ErbB2	[73]	retinoblastoma protein	[74]
Insulin-like growth factor -II	[75]	Tumour suppressor EXT proteins	[76]
P43	[77]	CYP-D	[60]
CLAVATA complex	[78]	Calcium-binding protein, Sorcin	[79]
Bile salt dependent lipase	[80]	Proteasome regulatory particle TBP7	[81]
Golgi apparatus casein kinase	[82]		
Cartilage oligomeric matrix protein (COMP)	[83]		
α1-antitrypsin ^1^	[84]		
Thyroglobulin	[85]		
HSV-1 glycoprotein B ^1^	[86]		
Apolipoprotein B	[87]		
Collagen	[88]		
Protein C ^1^	[89]		
Protein apparatus casein Kinase	[82]		
TLR 1,2,4	[90]		
Ig heavy chain and light chain	[91]		
Ig α chain	[92]		
Integrins	[90]		
Thrombospondin	[93]		

^1^ Indicates mutant forms of the protein.

**Table 4:** HSP90 protein clients and their contribution to carcinogenesis [7,44,188].

Protein Client	Role in Carcinogenesis
Receptor tyrosine kinases, serine/threonine kinases, steroid hormone receptors	Uncontrolled proliferation
Telomerases	Immortalization
AKT, NF-κB, p53, c-MET, Apaf-1, Survivin	Anti-apoptotic
HIF1α, VEGFR, PI3K/AKT, RTKs, flt-3	Angiogenesis
IRAK3	Escaping immune destruction
ARNT, ARRB1, HIF-1α, HMG1, SREBF1	Modification of cellular energetics
FANCA, MAFG, NEK8, NEK9, NEK11	Genome instability and mutation
IL-6, IL-8, IRAK1, IRAK2, IRAK3	Tumor-promoting inflammation
Plk, Wee1, Myc1, CDK4, CDK6, Myt1	Evading growth suppressors
MMP2,c-MET, SSDF-1	Invasion and metastasis

## Abbreviations

Aha1	Activator of Hsp90 ATPase protein 1
Apaf-1	Apoptotic protease-activating factor 1
ARNT	Aryl hydrocarbon receptor nuclear translocator
ARRB1	Beta-arrestin-1
Cdc37	Hsp90 co-chaperone Cdc37, cell division cycle 37
CDK4	Cyclin-dependent kinase 4
CHIP	Carboxyl terminus of Hsp70-interacting protein
CLAVATA	Receptor-like protein CLAVATA
c-MET	Proto-oncogene c-Met, hepatocyte growth factor receptor
CYP-D	Cyclophilin or Peptidyl-prolyl cis-trans isomerase F, mitochondrial
ERBB2	Receptor tyrosine-protein kinase erbB-2
FANCA	Fanconi anemia group A protein
FKBP51 and FKBP52	Peptidyl-prolyl cis-trans isomerase or 51 kDa FK506-binding protein
Flt-3	Receptor-type tyrosine-protein kinase FLT3
HIF1α	Hypoxia-inducible factor 1-alpha
HIP	Hsc70-interacting protein
HMG	High mobility group protein
HOP	Co-chaperone Hsp70/Hsp90-organizing protein
HSP	Heat shock protein
IRAK	Interleukin-1 receptor-associated kinase
MAFG	Transcription factor MafG
MHC class II	Major Histocompatibility Complex Class II
MMP2	Matrix metalloproteinase-2
Myt1	Membrane-associated tyrosine- and threonine-specific cdc2-inhibitory kinase
NEK8	Serine/threonine-protein kinase Nek8, Never in mitosis A-related kinase 8
NF-κB	Nuclear factor NF-kappa
p23	Co-chaperone protein 23
P53	Cellular tumor antigen p53
PI3K	Phosphatidylinositol 3-kinase
Plk	Serine/threonine-protein kinase PLK1
PP5	Serine/threonine-protein phosphatase 5
RTK	Receptor Tyrosine Kinase
Sgt1	Protein SGT1 homolog, Suppressor of G2 allele of SKP1 homolog
SREBF	Sterol regulatory element-binding protein
SSDF-1	Stromal cell derived factor -1
TLR	Toll-like receptor
TNF	Tumor Necrosis Factor
Tom70	Mitochondrial import receptor subunit TOM70
VEGFR	Vascular endothelial growth factor receptor
Wee1	Wee1-like protein kinase
WISp39	Waf1 Cip1 stabilizing protein 39
